# Quantitative research assessment: using metrics against gamed metrics

**DOI:** 10.1007/s11739-023-03447-w

**Published:** 2023-11-03

**Authors:** John P. A. Ioannidis, Zacharias Maniadis

**Affiliations:** 1 Departments of Medicine, of Epidemiology and Population Health, of Biomedical Data Science, and of Statistics, and Meta-Research Innovation Center at Stanford (METRICS), Stanford University, SPRC, MSOB X306, 1265 Welch Rd, Stanford, CA 94305 USA; 2 SInnoPSis (Science and Innovation Policy and Studies) Unit, Department of Economics, University of Cyprus, Nicosia, Cyprus; 3 Department of Economics, University of Southampton, Southampton, UK

**Keywords:** Citations, Bibliometrics, Research assessment, Gaming, Gift authorship, Self-citations, Fraud, Impact factor

## Abstract

Quantitative bibliometric indicators are widely used and widely misused for research assessments. Some metrics have acquired major importance in shaping and rewarding the careers of millions of scientists. Given their perceived prestige, they may be widely gamed in the current “publish or perish” or “get cited or perish” environment. This review examines several gaming practices, including authorship-based, citation-based, editorial-based, and journal-based gaming as well as gaming with outright fabrication. Different patterns are discussed, including massive authorship of papers without meriting credit (gift authorship), team work with over-attribution of authorship to too many people (salami slicing of credit), massive self-citations, citation farms, H-index gaming, journalistic (editorial) nepotism, journal impact factor gaming, paper mills and spurious content papers, and spurious massive publications for studies with demanding designs. For all of those gaming practices, quantitative metrics and analyses may be able to help in their detection and in placing them into perspective. A portfolio of quantitative metrics may also include indicators of best research practices (e.g., data sharing, code sharing, protocol registration, and replications) and poor research practices (e.g., signs of image manipulation). Rigorous, reproducible, transparent quantitative metrics that also inform about gaming may strengthen the legacy and practices of quantitative appraisals of scientific work.

## Introduction

Quantitative bibliometric indicators have become important tools for research assessments. Their advent has elated, frustrated, and haunted investigators, institutions and funding organizations. It is well documented that metrics have both strengths and limitations and that they need to be used with due caution. For example, the Leiden manifesto summarizes such a cautious, judicious approach to the use of bibliometric and other scientometric indicators [1].

However, misuse and gaming of metrics are rampant [2, 3]. The urge to “publish or perish” (or even “get cited or perish”) creates an environment where gaming of metrics is amply incentivized. A whole generation of old and new tricks try to make CVs and their impact look good and impactful—better and more impactful than they really are. Many of these gaming tricks can reach extravagant levels, as in the case of paper mills, massive self-citations, or citation cartels [4–6].

Concurrently, there are several efforts to try to improve metrics and make them available for all scientists, authors, and papers in ways that allow for proper standardization and more legitimate use [7–9]. Healthy efforts in bibliometrics and scientometrics should try to counter gaming and flawed practices. In the same way as antivirus software can detect and eliminate software viruses, proper metrics may be used to detect and correct for flawed, biased, gamed metrics. This review examines several gaming practices, including authorship-based, citation-based, editorial-based, and journal-based gaming as well as gaming with outright fabrication. We show how quantitative metrics may be used to detect, correct and hopefully pre-emptively diminish the chances of gaming and other flawed, manipulative research practices. Such an approach may help improve more broadly the standards of worldwide conducted research.

### Authorship-based gaming

Authorship of a scientific paper carries credit, responsibility, and accountability. Unfortunately, responsibility and accountability are often forgotten and the focus is placed on how to get maximal credit out of large numbers of coauthored publications (Table 1). Gift authorship (honorary authorship) refers to the phenomenon where authors are listed who have not made a sufficient contribution to the work that would justifiably deserve authorship [10, 11]. The Vancouver criteria make specific requests for the type of contributions that are necessary for authorship credit. However, it is likely that violations of these criteria are frequent. The exact frequency of gift authorship is difficult to pinpoint, but several surveys suggest high prevalence [12–20]. Estimates from such surveys may even be gross underestimates, since disclosing and/or admitting gift authorship is a risky disclosure. Gift authorship particularly thrives with specific researcher profiles and situations. The classic stereotype is the department chair placed as an author (often as the senior author) in most/all publications issued from that team, regardless of the level of contribution.

Gift authorship may co-exist with ghost authorship [21–25], where the author(s) who really wrote the paper do not even appear, while one or more gift authors take their place. The classic stereotype is when industry employees do the work and draft manuscripts published with names of academic gift authors, while the industry employees are invisible ghosts. Ghostwriting aims to confer academic prestige to the paper and minimize the perception of sponsor bias.

The advent of the concept of contributorship [26] has helped to allow provision of more granular information on the type of contributions made by each listed author in a scientific article. Moreover, efforts at standardization of contribution types, in particular the CREDIT system [27, 28] may allow some fairer appraisal of contributions. In theory, quantitative approaches can process and analyze in massive scale contributorship from each scientist across his/her papers and place these against the distribution of similar values from other authors in the same field. However, this is meaningful and feasible for papers with standardized (or at least comparable) credit types. More importantly, credit allocation may be gamed in the same way as plain authorship [29]. There is hardly any way to verify if the listed contributions are genuine, let alone at what level they occurred. Therefore, one may use information on authorship to understand whether some scientists exhibit unusual behavior suggestive of gaming.

In particular, large-scale bibliometric databases [30] have allowed the detection of hyper-prolific scientists, e.g., those with more than one full article published every 5 days (excluding editorials, commentaries, notes, and letters). This pattern may be particularly suspicious of loose authorship criteria especially when there are massive changes in the productivity of scientists linked to assumption of powerful positions, e.g., one can track that a scientist was in the 20th percentile of productivity in his field, but then moved to the top-0.01% after becoming a powerful administrator (unlikely to have much time for doing research).

In some teams and institutions, inordinate credit does not reflect on a single person, but may diffuse across many team members. This may be common in situations where multi-center work is involved with authorship awarded to many members from each of the participating components or local teams. There is an issue of balance here. On the one hand, credit needs to be given to many people, otherwise those left out would be mistreated. Mistreatment is common and often has other structural societal inequities as contributing factors (e.g., gender bias) [31]. On the other hand, there may be over-attribution of authorship to too many people, i.e., thin salami slicing of credit. The unfairness becomes more obvious when scientists from a team that over-attributes credit for authorship compete with scientists from teams that are less generous with authorship credit (or are even inappropriately not offering such credit). For example, for the same amount of work, one epidemiological consortium may decide to list 100 authors, while another one may list only 10, and a third one may list only 3.

Quantitative approaches can help sort out the co-author network of each author. They can generate citation metrics that account for co-authorship [32–34] and/or author position and even for relative contributions (if such information is available) and field-specific practices [35]. Therefore, two authors may have the same number of authored papers, but they may differ markedly in their relative placement and co-authorship patterns in these papers: one may have many papers as a single author or with few co-authors, while the other may routinely have 50 or more co-authors. On the other hand, they may have the same H-index for overall citations [36] but they may differ many-fold in a co-authorship-adjusted index, such as Schreiber’s hm index [31].

### Citation-based gaming

Many flawed evaluation systems still emphasize numbers of publications, while this measure should not matter in itself. A good scientist may publish one, few, many or huge number of papers. What should matter is their impact, and citation metrics are used as surrogates of impact. However, these measures can also be gamed, and different metrics differ in their gaming potential.

First, publishing more papers may lead to more citations by itself. Citations are not fully rational, and many scientists cite papers without even having read them [37]. While some papers are never cited, this proportion has probably decreased over time and the frequent quote that half of the papers are never cited is a myth [38]. One may penalize publishing large numbers of papers and some have even argued that there should be a cap on how many total words a scientist can publish [39]. Such penalizing and capping is ill advised, however. It may intensify selective reporting and publication bias, as scientists would struggle to publish only extreme, nice-looking results that may attract more attention. It is probably more appropriate not to pay any attention to the number of publications (except for the extreme tail of hyper-prolific authors) and allow scientists to disseminate their work in whatever way and volume they deem most appropriate. However, one may examine other quantitative metrics such as citations per paper, and place these metrics in a percentile ranking against papers from the same field, e.g., a scientist may have 100 publications and 1000 citations and be at the 25th percentile of his field for citations per paper (1000/100 = 10). Another scientist may also have 1000 citations, but with 1000 publications may be at the bottom 0.1% percentile for citations per paper (1000/1000 = 1), suggesting he/she is publishing very trivial work.

Self-citation is a classic mechanism that increases one’s citation count [40, 41]. Self-citations can be defined in different ways. A strict definition includes references to one’s own work. A more inclusive definition includes also references to one’s work by any of the co-authors of that work. Many self-citations are entirely appropriate [42]. Science requires continuity and linking of the current work to relevant previous work. In fact, avoidance of such linking and not use of self-citations would be inappropriate and even ethically reprehensible—e.g., it may mislead that some work is entirely novel, and/or could lead to undeclared self-plagiarism [43]. Self-citations may also have both a direct effect in increasing total citations and an indirect effect—when a work is mentioned more frequently, other scientists may notice it and cite it as well [44] (Table 1).

**Table 1:** Examples of gaming behaviors and how quantitative metrics may help

Gaming behavior	How quantitative metrics can help
Authorship-based	
Massive authorship of papers without meriting credit (gift authorship)	Detection of hyper-prolific scientists, especially those with massive changes in their productivity linked to assumption of powerful positions
Team work with over-attribution of authorship to too many people (salami slicing of credit)	Use of citation metrics that account for co-authorship, evaluation of provenance of publications and citations that reflect team work in large teams that routinely use massive authorship lists
Citation-based	
Massive self-citations	Detection of outliers in the distribution of the proportion of self-citations within the same scientific subfield
Citation farms	Analysis of the provenance of citations showing that they come from a narrow circle of authors, often cross-citing one another
H-index gaming	Ratio of total citations over the square of H-index is very low; also often linked to some of the behaviors above
Editorial-based	
Editorial nepotism	Detection of extreme numbers of papers published in a specific journal, often in association with holding an editor-in-chief position in that journal
Journal-based	
Journal impact factor gaming	Multiple processes and gaming tools can be detected by bibliometric analysis at the level of journal self-citation and co-citation patterns; it may also involve gaming gains for specific researchers as well
Outright fabrication	
Paper mills and spurious content papers	Mapping of paper mill products across scientific publications, identification of papers with content that is incompatible with the mission of a journal
Spurious massive publications for studies with demanding designs	Detection of sudden, massive production of papers with demanding study designs for which a scientist or team have no prior tradition and resources to run, e.g., massive sudden production of randomized trials in some institutions in less developed countries

Self-citations would require an impossibly strenuous in-depth evaluation to examine whether each of them is inappropriate or not. However, centralized bibliometric databases [5, 45] can allow placing the proportion of self-citations for an author as a percentile ranking against the self-citations of other authors in the same scientific field. Extreme outliers (adjusting for field and possibly also age [5]) may be characteristic of gaming behavior (Table 2).

**Table 2:** Proportion of self-citations (PSS) among the total number of citations received: 1% and 5% threshold among top-cited established authors in different scientific fields

Field	Authors	PSS top 5% threshold (%)	PSS top 1% threshold (%)
Agriculture, fisheries and forestry	313,703	27.25	36.07
Built environment and design	48,681	26.44	35.98
Enabling and strategic technologies	793,331	32.00	45.92
Engineering	708,314	34.26	48.12
Information and communication technologies	646,368	31.40	49.79
Communication and textual studies	43,349	17.75	27.27
Historical studies	42,082	26.68	36.43
Philosophy and theology	23,638	21.28	29.64
Visual and performing arts	6333	22.72	32.94
Social sciences	191,880	19.48	28.92
Biomedical research	737,571	25.16	33.80
Clinical medicine	2,886,526	22.11	29.31
Psychology and cognitive sciences	112,843	22.68	30.48
Public health and health services	169,053	21.08	28.66
Biology	332,772	28.32	39.07
Chemistry	645,810	32.17	45.56
Earth and environmental sciences	302,410	29.76	39.01
Mathematics and statistics	112,257	37.71	55.97
Physics and astronomy	785,346	40.60	52.69
Economics and business	168,428	16.58	24.18
Unassigned	427		
Total	9,071,122	29.44	42.42

Data are from https://elsevier.digitalcommonsdata.com/datasets/btchxktzyw/5 with citation information until the end of 2021 for the whole career of scientists. The 9 + million considered authors are those with at least 5 publications of full papers (articles, reviews, conference papers). Thresholds differ markedly across age groups (young scientists who started publishing recently have a median proportion of self-citations that can be threefold higher than that of very senior authors) [5]. The thresholds given here are based on the 2% top-cited authors in each field according to a composite citation indicator [7], thus providing a reference against a cohort of influential established investigators that may be as close as possible to a “gold standard”. Self-citations here are defined as those citations to a publication that come from the author being evaluated or his/her co-authors in that same paper. For example, in the Agriculture, Fisheries and Forestry field, 5% of the top-cited authors have > 27.25% of their citations be self-citations and 1% have 1% of the top-cited scientists have > 36.07% of their citations be self-citations

Self-citation practices may take also complex forms. Occasionally, the authors may collude to cite each other’s works, even though they are not co-authors. Such citation cartels (citation farms) usually involve a small number of authors. Flow of citations may not necessarily be equally towards all members of the cartel. For instance, one or a few members may be cited, while the others may enjoy other repayments. The members of the citation farm may be in different institutions and countries. Again, quantitative metrics is the best way to diagnose a cartel. Usually, a large number of scientists cite one author’s work and citations from each citing author account for a very small portion of the total citations. Conversely, in a citation farm, a handful of citing authors may account for > 50% or even > 80% of the citations received.

Some of the inappropriate self-citing or citation farming behavior may even aim to inflate selectively some specific citation metric considered most important. For example, the Hirsch h-index has enjoyed inordinate popularity since its introduction in 2005 [35]. H-index can be more easily gamed than the number of total citations. Self-citers preferentially (“strategically”) cite papers that readily boost the H-index [44]. Again, quantitative metrics can help detect this behavior, for instance by examining the ratio of total citations over the square of the H-index. Average values for this ratio are about 4 [35]. Very small values suggest that citations have been targeted to papers that boost the H-index while total citations are relatively more difficult to manipulate.

### Editorial-based gaming

Journals may not treat equally all the authors who submit their work to them. Some authors may be favored. Proportion of submissions accepted may vary markedly across authors. Often this is entirely normal: some scientists truly submit better work than others. However, difficulties arise when the submitting and publishing authors are directly involved with the journal, as editors-in-chief or as staff members. With the rapid proliferation of journals, including mega-journals [46], the numbers of editors-in-chief, guest editors and staff members has also increased markedly.

Editors are fully entitled (and even encouraged as part of their job) to write editorials and commentaries on topics that they consider important. This activity is fully legitimate. These editorial pieces may go through no or very limited review and get published quickly on hot matters. Some high-profile journals, such as Nature, Science, and BMJ have numerous staff writers and science journalists (as staff or free lancers) who write news and feature stories, often massively. An empirical analysis [47] has shown that some of these writers have published 200–2000 papers in these venues where a scientist would consider a career milestone to publish even a single article. Most of these authors are usually not competing in the world of academia. However, exceptions do occur where editorialists publishing massively in one journal may be academics. Other editors may give up their editorial career at some point and move to competitive academia. Another concern is that these editorial publications often have no disclosures of potential conflicts of interest [47]. Some editors have great power to shape science narratives in good or bad ways. Quantitative metrics can separate the impact of authors due to non-peer-reviewed editorial material versus peer-reviewed full articles.

A more contentious situation arises when an editor-in-chief publishes original full articles in his/her own journal. While this is possible and not reproachable if done sporadically (especially if the paper is handled by another editor), some authors raise concerns about this practice, when it is common. Empirical analyses have shown the prevalence of editorial nepotism practices [48]: in a survey of 5,468 biomedical journals, 5% of the journals had > 10.6% of their published articles authored by a single person, typically the editor-in-chief and/or other highly preferred members of the editorial board. Quantitative analyses can map the distribution of papers of an author across different journals and identify if there is an inordinate concentration of full, original papers in journals where the author is editor-in-chief.

### Journal-based gaming

Most of the gaming at the level of journals involves efforts to boost the journal impact factor [49]. Detailed description of the multiple well-known problems and gaming practices for this metric is beyond the scope of this paper. Nevertheless, many of the gaming practices used for single scientists have equivalents for gaming at the journal level, e.g., coercive journal self-citation (requests by the editor to cite other papers published by the journal) and citation cartels involving journals rather than single authors (“citation stocking”) [50]. Multiple processes and gaming tools can be detected by bibliometric analysis at the level of journal self-citation and co-citation patterns. Journal impact factor manipulation may also involve gaming gains for specific researchers as well, in particular for the editors, as described above. Journals with higher impact factors get cited more frequently, even when it comes to papers that are identically published in other journals (e.g., reporting guideline articles) [51].

### Gaming with outright fabrication

The gaming practices described so far typically do not have to involve fabrication. The gamed published and cited material is real, even though its quality may be suboptimal, given the inflated productivity. However, there are also escalating gaming practices that involve entirely fabricated work.

In paper mills, a for-profit company produces papers (typically fraudulent, fabricated ones), which it sells to scientists who want to buy authorship slots in them. The papers are for sale before submission or even after acceptance [52–54]. An increasing proportion of retractions in the last 7 years has been for paper mill-generated articles [55]. It is unknown though whether this may be just the tip of the iceberg and these retracted papers are those where the fabrication is more egregious and thus readily discernible. The advent of more powerful large language models may make the paper mill products more sophisticated and difficult to identify [56]. Software is evolving to detect use of such large language models, but it is unclear whether such detection software would be able to catch up. Involvement of artificial intelligence in writing scientific papers is an evolving challenge for both genuine and fraudulent papers. Several journals have tried to tackle this challenge, but reactions have not been uniform [57–60].

There are many other egregious evolutions in the publishing world, a consequence of publish-or-perish pressure. Predatory journals (journals publishing content for a fee but practically without peer review) are widely prevalent, but their exact prevalence is difficult to ascertain, given the difficulty to agree on which journals are predatory [61–63]. Some of the most notorious phenomena are hijacked journals and publication of spurious content. Hijacking happens when a site belonging formerly to a discontinued serious journal is taken over by a predator who uses the name and the prestige of the previous journal for operating the predatory business [64]. Some papers also get published in journals with totally unrelated aims/mission/subject matter coverage; such spurious content is indication for fraudulent behavior (e.g., may be associated with both paper mills and predatory publishing).

Again, bibliometric, quantitative indicators can be used to place the prevalence of such behaviors in publication corpora of single authors, teams, institutions, and journals into perspective. Indicators may include frequency of documented paper mill products, hints of inappropriate use of large language models, hints of predatory or other inappropriate journal behavior (e.g., percentage of papers published in journals that lost their journal impact factor), and percentage of papers with content unrelated to the other published content of the journal.

Even in very serious journals, the proportion of fabricated papers may be increasing over time. John Carlisle, an editor of a prestigious specialty journal (Anesthesia) requested the raw data of over 150 randomized trials submitted to his journal and concluded that in 30–40% of them the data were so messed up and/or improbable that he called these trials zombie trials [65, 66]. Zombie trials tend to come from particular institutions and countries. Such trials have demanding clinical research designs that are difficult to perform, let alone perform massively. Quantitative bibliometric analysis can allow the detection of sudden, massive production of papers with demanding study designs for which a scientist or team have no prior tradition and resources to run, e.g., massive sudden production of randomized trials in some institutions in less developed countries [67].

### Metrics of best research practices and of poor research practices

Most bibliometric and scientometric indicators to-date have focused on counting numbers and citations of scholarly publications. However, it is very important to capture also information on research practices, good and bad. These research practices may in fact often be well reflected in these publication corpora. For example, good research practices include wide data sharing, code sharing, protocol registration, and replications. It is currently possible to capture for each scientist how often he/she used these standards in his/her published work. For example, a free, publicly available resource covers all the open-access biomedical literature for these indicators [68].

It is also possible to capture systematically the use of several poor research practices. For example, image manipulation is a common problem across many types of investigation. There are already appraisal efforts that have tried to generate data on signs of image manipulation across large publication corpora rather than doing this exercise painstakingly one paper at a time [69, 70].

Another potential sign of poor research practices is retractions. At least one science-wide assessment of top-cited scientists currently excludes from consideration those with retracted papers based on the inclusive Retraction Watch database (https://retractionwatch.com/2022/11/15/why-misconduct-could-keep-scientists-from-earning-highly-cited-researcher-designations-and-how-our-database-plays-a-part/). The majority of retractions may signal some sort of misconduct. However, in a non-negligible proportion of cases, they may actually signal honest acknowledgment of honest error—a sign of a good scientist that should be praised and encouraged if we wish to see better self-correction in the scientific record. Therefore, when retractions are present, they need to be scrutinized on a case-by-case basis regarding their provenance and rationale. Making wider use of available resources, such as the Retraction Watch database, and improving and standardizing the retraction notices [71] may help add another important dimension to research appraisals.

### Putting it together

Table 3 lists a number of quantitative metrics and indicators that are currently readily available (or can be relatively easily obtained) from centralized databases. The examples of scientists shown are entirely hypothetical and do not correspond to specific real individuals; they are provided for illustrative reasons. All three scientists are highly cited, in the top 1.8%, 0.9% and 0.7% of their scientific domain, respectively. However, two of the three scientists show problematic markers and/or score very low for markers of transparency and reproducibility.

**Table 3:** An illustrative list of quantitative metrics and indicators to appraise a scientist

Metric/indicator	Example 1	Example 2	Example 3
Main scientific subfield (% of papers)	Rheumatology (56%)	Epidemiology (60%)	Cardiovascular (85%)
Secondary scientific subfield (% of papers)	Allergy (22%)	Oncology (26%)	General medicine (10%)
Ranking of composite citation indicator in the main scientific subfield	Top 1.4%	Top 0.9%	Top 0.7%
Proportion of self-citations	8.2%	38.3%^b^	16%
Ranking of proportion of self-citations in the main scientific subfield	27th percentile	99th percentile (top 1%)^b^	52nd percentile
Hyperprolific publication behavior, e.g., any year with more than 72 full papers	No	No	Yes^b^
Number of co-authors with whom the scientist has published over 50 papers	3	84^b^	50^b^
Ratio of total citations over H^2^	4.1	3.8	3.2
Any evidence for citation farm	No	No	Yes^b^
Proportion of citations to non-full papers^a^	20%	15%	6%
Number of retracted papers	0	2^b^	0
Number of detected paper mill papers	0	0	0
Percentage of papers published in journals that lost their impact factor	2.4%	14.2%^b^	1.7%
Proportion of open-access papers	42%	14%^c^	40%
Proportion of papers with data sharing	15%	2%^c^	10%
Proportion of papers with code sharing	5%	0%^c^	3%
Proportion of papers with protocol registration	6%	1%^c^	0%^c^

The examples are entirely hypothetical and do not represent specific real scientists, they are presented for illustrative purposes. Most of the listed metrics can be readily obtained from centralized, publicly available analyses, e.g., the Scopus-based citation databases of composite citation indicators that also include self-citation metrics and percentiles [7, 45]; available databases (see Retraction Watch Database User Guide—Retraction Watch) of retractions for number of retracted papers (and reasons such as paper mills); PubMed (proportion of open-access papers); and the publicly available algorithms for data sharing, code sharing and registration in the open-access literature [68]. The remaining listed metrics can be currently obtained from subscription-based bibliometric databases (e.g., Scopus), but they should also be readily possible to standardize and make publicly available. Percentile thresholds (especially for critically poor values, e.g., worse 5% and worse 1%) would be useful to make publicly available for all of these metrics, ideally field-adjusted

^a^Full papers include articles, reviews, and conference papers

^b^Problematic metric

^c^Good research practice with frequency substantially below typical values for scientific domain

Efforts should be devoted to make such datasets more comprehensive, covering routinely such indicators across all scientific investigation, and with percentile rankings adjusted for scientific field. Each metric should be used with full knowledge of its strengths and limitations. Attention should focus particularly on extreme outliers; modest differences between two authors should not be seen as proof that one’s work is necessarily superior to another. Even with extreme values, metrics should not be used to superficially and hastily heroize or demonize people. For example, very high productivity may reflect some of the best, committed, devoted scientists; some recipients of massive gift authorships; and some outright fraudsters. While single metrics may not suffice to fully reliably differentiate these groups, the complete, multi-dimensional picture usually can clearly separate who is who.

## Data Availability

Not applicable.
